# RASAL2 mediated the enhancement of YAP1/TIAM1 signaling promotes malignant phenotypes of pancreatic ductal adenocarcinoma

**DOI:** 10.7150/ijbs.72204

**Published:** 2022-06-27

**Authors:** Yangyang Yue, Kaijie Wu, Weikun Qian, Zeen Zhu, Simei Zhang, Wunai Zhang, Weifan Zhang, Shuai Wu, Li Li, Zheng Wu, Qingyong Ma, Keping Xie, Zheng Wang

**Affiliations:** 1 Department of Hepatobiliary Surgery, First Affiliated Hospital of Xi'an Jiaotong University, Xi'an, Shaanxi 710061, China.; 2 Department of Vascular Surgery, First Affiliated Hospital of Xi'an Jiaotong University, Xi'an, Shaanxi 710061, China.; 3 Department of Urology Surgery, First Affiliated Hospital of Xi'an Jiaotong University, Xi'an, Shaanxi 710061, China.; 4 Department of Ophthalmology, First Affiliated Hospital of Xi'an Jiaotong University, Xi'an, Shaanxi 710061, China.; 5 Center for Pancreatic Cancer Research, South China University of Technology, Guangzhou, Guangdong 510006, China.

**Keywords:** RASAL2, YAP1, TIAM1, PDAC, progression

## Abstract

Pancreatic ductal adenocarcinoma (PDAC) is characterized by a high incidence of metastasis and dismal prognosis. As a member of Gas-Gap gene, RASAL2 is involved in the hydrolysis of RAS-GTP to RAS-GDP and abnormal expression in human cancers. Here we firstly described the function of RASAL2 on PDAC to enrich the knowledge of RAS family.We interestingly observed that RASAL2 expression was upregulated in PDAC at both mRNA and protein levels, and high expression of RASAL2 predicted a poor prognosis in PDAC patients. Additionally, RASAL2 promoted malignant behaviors of PDAC in vitro and in vivo. To determine the mechanistic roles of RASAL2 signaling and its potential as a therapeutic target in PDAC, we clarified that RASAL2 could accumulate the TIAM1 expression in different level through inhibiting YAP1 phosphorylation, increased TIAM1 mRNA expression and suppressed ubiquitination of TIAM1 protein. In conclusion, RASAL2 enhances YAP1/TIAM1 signaling and promotes PDAC development and progression.

## Introduction

Pancreatic cancer incidence and mortality rates have increased in the past decade, and the five-year survival rate is less than 10% 1. Pancreatic ductal adenocarcinoma (PDAC) is the most common pathological type of pancreatic cancer 2. More than 50% of PDAC patients have had distant metastasis at the time of diagnosis. Although a minority of patients can undergo surgical resection, most of them will develop metastases rapidly after surgery 3, 4. To develop more sensitive and specific methods for early PDAC diagnosis and more effective therapeutics, better understanding of the molecular basis of PDAC is essential and urgent.

Due to the relation between k-ras mutation and pancreatic cancer, the Ras family is paid more attention. As a member of RAS GTPase-activating protein family (RAS-GAP), RAS protein activator like 2 (RASAL2) was first reported as a tumor suppressor and down-regulated in different types of tumors, *e.g.*, luminal B breast cancer, lung cancer, and ovarian cancer 5-12. It is located at chromosome 1q25.2 in humans 13. RASAL2 is catalyzes the hydrolysis of RAS-GTP to RAS-GDP 7, 13. However, the oncogenic roles of RASAL2 were reported recently in some solid tumor such as colorectal cancer, liver cancer and triple-negative/estrogen receptor-negative breast cancer 14-18.

As an important RAS-GAP gene, it is necessary to clarify the role on pancreatic cancer. In this study, we sought to determine the role of RASAL2 in PDAC and its relation with malignant behavior. To clarify the possible mechanism could contribute to understanding the molecular mechanisms of PDAC development and progression, and identify potential molecular targets for designing novel approaches to PDAC diagnosis and treatment.

## Methods and Materials

### Cell culture and reagents

Human PDAC cell lines PANC-1, MiaPaCa-2 were purchased from the Chinese Academy of Sciences Cell Bank of Type Culture Collection (CBTCCCAS, Shanghai, China). The PANC-1 and MiaPaCa-2 were cultured at 37 ˚C with 5% CO_2_ in Dulbecco's modified Eagle's medium (DMEM; Gibco; Thermo Fisher Scientifc, USA) supplemented with 10% fetal bovine serum (FBS).

### The sources of antibodies

Antibodies against RASAL2 (Rabbit, Abcam, Cambridge, UK; Mouse, Santa Cruz Biotechnology, CA, USA; Rabbit, Proteintech, Manchester, UK); TIAM1 (Mouse, Santa Cruz Biotechnology, CA, USA; Sheep, R&D Systems, MN, USA); Ubiquitin, p-YAP1 and YAP1 (Rabbit, Abcam, Cambridge, UK); E-cadherin, Vimentin, MMP2 and Claudin1 (Rabbit, Cell Signaling Technology, Danvers, Ma, USA); β-actin and GAPDH (Mouse, Proteintech, Manchester, UK) were used for immunohistochemistry and western blot analyses.

### Clinical specimens and immunohistochemistry

All the PDAC and normal tissues were collected from the Department of Hepatobiliary Surgery, The first Affiliated Hospital of Xi'an Jiaotong University, Xi'an, China. The Collection and use of clinical samples was approved by the Ethical Committee of Hospital and informed consent was obtained from all patients. The tissue sections were deparaffinized in 60˚C for 4h and immersed in xylene quickly, rehydrated in a series of grade alcohols. After washing 3 times by 5 minutes once with PBS buffer (exclusive of potassium), tissue sections were subjected to 5-min pressure cooker antigen retrieval methods, 10-min of quenching endogenous enzyme. Next, the primary antibody was incubated at 4˚C overnight, and Dako Cytomation EnVision-HRP reagent was incubated for 30 mins at room temperature after washing with PBS. Then, diaminobenzidine (DAB) was added to detect the signals and hematoxylin to stain nucleus. The intensity of staining was evaluated by image J software (NIMH, National Institutes of Health, USA).

### Western blot analysis

The cell lysates were prepared using RIPA buffer (50 mM Tris, PH 8.0, 150 mM NaCl, 0.1% SDS, 1% NP40 and 0.5% sodium deoxycholate) containing proteinase inhibitors (1% inhibitors cocktail and 1 mM PMSF) (Roche Applied Science, Germany) for 10 min in ice and centrifuged at 12000g for 15 min at 4°C. The samples were separated by SDS-PAGE and transferred to polyvinylidene fluoride (PVDF) membranes. After blocking with 5% bovine serum albumin, the membranes were incubated with primary antibody at 4°C overnight. Next, the membranes were washed with TBST buffer for 3 times and incubated with peroxidase-conjugated secondary antibody for 1h at room temperature. After washing with TBST buffer for 3 times again, the membranes were visualized by using an ECL chemiluminescent detection system (Bio-rad, USA).

### Plasmid transfection and lentiviral infection

RASAL2 short hairpin RNA (shRNA) was used to knockdown RASAL2 in PDAC cells. The plasmids pGPH1 contained the shRNA of RASAL2 were purchased from GenePharma (Shanghai, China), and lentiviral system pLKO.1 (Oligoengine, Seattle, WA) was used to construct RASAL2 shRNA expression vector. Lentiviral RASAL2 was obtained from GeneCopoeia (Guangzhou, China). After 48h of transfection with 8 μg/ml polybrene following the manufacturer's instructions, the cells were harvested for further experimentation.

### RNA extraction and quantitative real-time polymerase chain reaction (qRT-PCR)

The total RNA was extracted from tumor cells by RNAfast 200 reagents (Fastagen Biotechnology, Shanghai, China) and from tumor tissues by TRIzol reagent (Thermo Fisher Scientific, Waltham, MA, USA). After quantitation by absorbance at 260 nm, the RNA samples were transcribed reversely by PrimeScript RT Master Mix (Takara Bio, Dalian, China). The quantitative PCR was performed by using SYBR-Green PCR Master Mix (Takara Bio, Dalian, China). The specific primers used were listed in Supplementary Materials.

### *In vitro* cell proliferation assays

For MTT assay, PANC-1 cells with RASAL2 knockdown and MiaPaCa-2 cells with RASAL2 overexpression were plated in 96-well plates at 1500 cells/well for 24h, 48h, 72h and 96h, then 20 µl MTT (Sigma-Aldrich; USA) was added into each well and the plates were cultured for another 4h at 37 ˚C with 5% CO_2_. The medium was removed and 150 µl DMSO was added into wells, and the optical density values were determined by microplate autoreader (Bio-Tek Instruments, Winooski, USA). For Colony formation assay, PANC-1 and MiaPaCa-2 cells were seeded into 6-cm dishes with 2000 cells/well for 2 weeks. Then dishes were washed by phosphate-buffered saline (PBS) for three times, fixed with 4% paraformaldehyde and stained with 1% crystal violet solution for 15 mins. The numbers of colonies were counted from three random fields. For cell cycle analysis, cells were washed with ice-cold PBS, and fixed in ice-cold 70% ethanol at 4°C. After removing the remaining PBS, cell cycle was quantified by the PI staining detection kit (Roche Applied Science, Germany). The stained cells were analyzed by FACSCalibur™ flow cytometer (BD Biosciences, USA).

### *In vitro* migration and invasion assays

PANC-1 cells with RASAL2 knockdown and MiaPaCa-2 cells with RASAL2 overexpression were plated in 6-well plates with cross marker lines on the back. When the cells reached 100% confluence, a 200 µl pipette tip was applied to make artificial wounds. The wounds healing was observed by using an inverted microscope after 12h and 24h at the same position, and the rate of wound healing was evaluated by the Image Pro Plus 6.0 software (Media Cybernetics company, USA). For transwell migration assay, 4×10^4^ tumor cells in 300 µl serum-free DMEM medium were plated into the upper chamber with 8-µM pore polycarbonate membrane filters (Millipore, USA). For invasion assay, 8×10^4^ tumor cells in 300 µl serum-free DMEM medium were added into the upper chamber inserts with Matrigel (BD Biosciences, USA) flatted for 4h in advance. After incubation for 24 h or 36 h, the upper inserts were fixed with 4% paraformaldehyde for 15 min and stained with 0.1% crystal violet for 15 min at room temperature. Then, the number of cells at lower side of the inserts was counted in three random fields by using an inverted light microscope at 100× magnification.

### RNA Sequencing

Total RNA of tumor cells was extracted using TRIzol reagent (Life Technologies, Inc., Gaithersburg, MD). The qualification, quantification, library preparation and RNA-sequencing of RNA samples were performed by Novogene Co., LTD (Beijing, China). Differentially Expressed Genes (DEGs) analysis and Gene set enrichment analysis (GSEA) were conducted by using edgeR and clusterProfiler packages. Corrected P < 0.05 and fold change > 2 were set as the threshold parameters.

### Double immunofluorescence assay

Cells were cultivated in circular glasses in 24-well plates after treatment, fixed in 4% paraformaldehyde for 15 min in advance, washed with PBS for three times, treated with permeabilization solution (1% Triton X-100 in PBS), washed with PBS again, and blocked with 5% bovine serum albumin (Sigma-Aldrich, Germany) for 1h. Samples were incubated with two primary antibodies (Rabbit anti-RASAL2, 1:100; Mouse anti-TIAM1 1:100) in PBS buffer overnight at 4°C. Next, samples were washed with PBS for three times and incubated with a mixture of secondary antibody (Alexa Fluor 488 and 594, 1:2000, Thermo Fisher Scientific) for 1h at room temperature, then stained by DAPI (1:5,000) for 15 min in the dark. Laser scanning confocal microscopy (Nikon A1R/A1) was used to evaluate the samples.

### Co-immunoprecipitation assay

Co-immunoprecipitation assay was performed by using Immunoprecipitation kit from Abcam (ab206996, Cambridge, UK) following the manufacturer's protocol. For ubiquitination analysis, the cells were incubated in 20 µM MG132 (MedChem Express, NJ, USA) for 8h before protein collection. About 500 μg of cell lysate was incubated with relevant antibodies or control IgG at 4 ℃ overnight, then 60 µl Protein A/G agarose was added. After rotating for 1h at 4 ℃, the immune complexes were washed for three times and were eluted for western blot analysis.

### ChIP assay

Magna CHIP^TM^ A/G Kit (MERCK, Darmstadt, Germany) was used to perform ChIP assay according to the manufacturer's instructions. Pan-TEADs (Rabbit, Cell Signaling Technology, Danvers, Ma, USA) was used to immunoprecipitate TEADs. DNA was purified and determined by polymerase chain reaction (PCR). The primers used to analyze the promoter region of TIAM1 were listed in Supplementary Materials.

### Tumor xenograft model

Female athymic BALB/c nu/nu mice about 4-6 weeks old were used for the experiment. The use of animals was approved by the ethical committee of Xi'an Jiaotong University. MIA PaCa-2 cells with RASAL2 overexpression (Lv-RASAL2) and control cells (Lv-NC) were prepared for further use. For subcutaneous xenotransplanted tumor model, six mice per group were randomly distributed into two groups (Lv-RASAL2 and Lv-NC), 100 µl serum-free DMEM medium containing Matrigel (1:1, v/v) and 1×10^6^ cells were injected subcutaneously into flanks of the mice. About 30 days after tumor cell injection, the mice were sacrificed and xenograft tumors were harvested, weighed and processed for immunohistochemistry staining. For tail-vein injection model of cancer metastasis, ten mice per group were randomly distributed into two groups (Lv-RASAL2 and Lv-NC), 2×10^6^ cells were suspended in 200 µl serum-free DMEM and injected via the tail vein. About 6 weeks after tumor cell injection, D-luciferin substrate (Biosynth, Naperville, IL, USA) in PBS with 450 mg/kg was injected into peritoneal cavity; then 15-20 minutes later, mice were anesthetized and bioluminescence imaging (BLI) was performed to detect the distant metastases in the lung and other organs.

### Bioinformatics and statistical analysis

The PDAC public datasets GSE15471, GSE28735 and GSE16515 were downloaded from the gene expression omnibus (GEO: https://www.ncbi.nlm.nih.gov/geo/) database. The RASAL2 expression data based on RNA-sequence was acquired from cBioPortal (www.cbioportal.org) for The Cancer Genome Atlas (TCGA). The samples without data were excluded from the analysis. The mRNA expression data was analyzed and performed by GraphPad Prism version 7.0 software (GraphPad Software, USA). All the statistical analyses were performed by SPSS 22.0 software. All data were reported as mean ± SD of triplicate experiments, and the differences between two groups were compared by the two-tailed Student's (t-test) or one-way analysis of variance. *P<0.05 was considered statistically significant.

## Results

### RASAL2 played an oncogene role in PDAC and predicted poor prognosis, possibly related with k-ras mutation

To understand the role of RASAL2 in PDAC development and progression, we firstly analyzed the deferentially expressed genes and identified top 30 overexpressed genes in PDAC from GSE15471 dataset of GEO database (based on Affymetrix Human Genome U133 Plus 2.0 array sequencing of pancreatic tumor and matched normal tissues). RASAL2 was one of 30 genes that significantly increased (**Supplement Fig. 1a**). In other two datasets of GSE28735 and GSE16515, we obtained similar results (**Supplement Fig. 1b**). RASAL2 mRNA expression also was elevated in PDAC from oncomine database and TCGA matched GTEx database (**Supplement Fig. 1c** and **1d**).

To further investigate the relation between RASAL2 and clinical characteristics of PDAC patient, we compared the expression of RASAL2 at different stage. The expression of RASAL2 mRNA at G2 and G3/4 stages was higher than G1, (**Supplement Fig. 1e**). As the **Supplement Table 1** shown, the expression of RASAL2 mRNA was correlated with tumor grade, tumor size and new tumor event. Additionally, patients with high expression of RASAL2 mRNA had much shorter overall survival (OS) and disease-free survival (DFS) than those with low expression of RASAL2 mRNA (**Supplement Fig. 1f**). Furthermore, analyses KRAS mutation indicated that patients with KRAS mutation had a higher mRNA expression of RASAL2 than those without (**Supplement Fig. 1g**). As one member of Ras Gap family, it was confirmed that RASAL2 was an oncogene for PDAC, possibly related with k-ras mutation.

**Figure 1:**
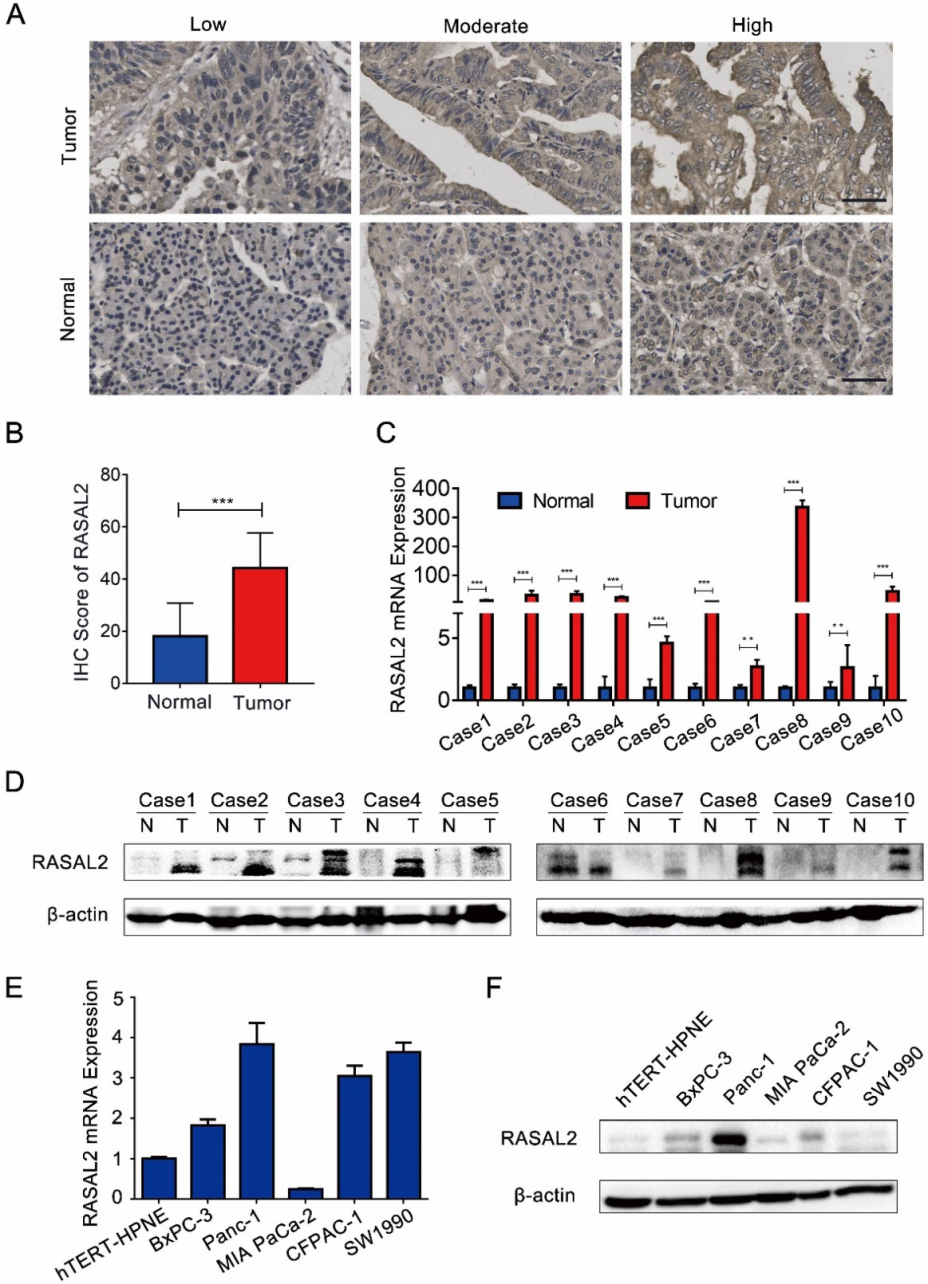
**RASAL2 expression in human PDAC tissue and cell lines**. **a** Representative immunohistochemistry (IHC) images of high, moderate and low RASAL2 staining in PDAC tissues (n = 70) and normal pancreas tissues (n = 20). **b** The quantification analysis of IHC score. **c** and **d** RASAL2 mRNA and protein expression in 10 human PDAC tissues. **e** and **f** RASAL2 mRNA and protein expression in human PDAC cell lines. 18S was applied as the endogenous control for quantitative real-time RT-PCR, and β-actin was used as a loading control for western blotting assays (N = 3). (***p < 0.001).

Compared with normal pancreas tissues, the expression levels of both RASAL2 mRNA and protein were upregulated in PDAC tissues (**Fig. 1a-d and Supplement Fig. 2a**). *In vitro*, RASAL2 expression was higher in PDAC cell lines than that in pancreatic duct epithelium cell line hTERT-HPNE (**Fig. 1e**-**f and Supplement Fig. 2b**). Therefore, overexpression of RASAL2 directly correlated with PDAC progression clinically and predicted poor patient survival.

### Modulation of RASAL2 influenced malignant behaviors of PDAC cells* in vivo and in vitro*

**Figure 2:**
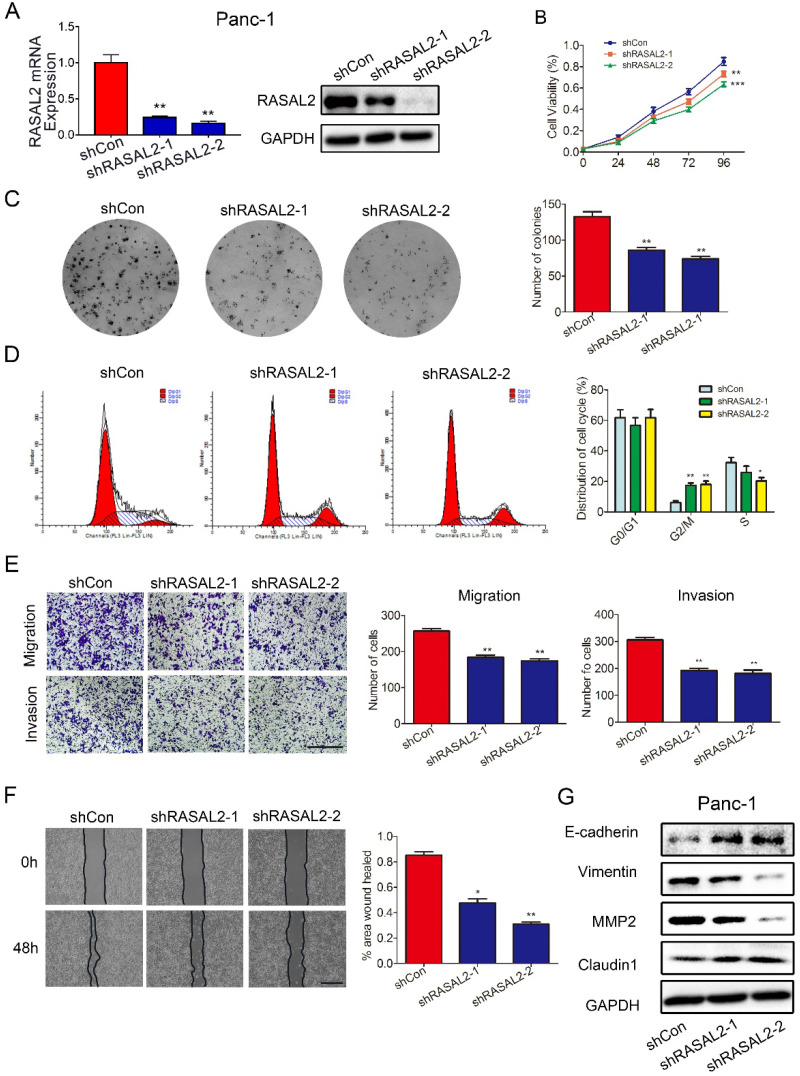
** Knockdown of RASAL2 inhibited PDAC cell growth and migration**. **a** Quantitative real-time RT-PCR and western blotting analysis of RASAL2 mRNA and protein expression in PANC-1 cells transfected with RASAL2 shRNAs or shControl. **b** and** c** MTT and colony formation assays showed that RASAL2 silence reduced the cell proliferation (right panel, quantitative analyses; N = 3). **d** Flow cytometric analysis detected cell cycle distribution via PI staining after RASAL2 silence (right panel, quantitative analysis; N = 3). **e** Representative images of Transwell migration and invasion assays in RASAL2-knockdown PANC-1 cells, quantification analysis was shown right (N = 3). **f** Representative pictures of wound healing in RASAL2 silenced PANC-1 cell line, quantification analysis was shown right (N = 3). **g** E-cadherin, Vimentin, MMP2 and Claudin1 were detected by western blot. 18S was used as the endogenous control for quantitative real-time RT-PCR, and GAPDH was used as a loading control for western blotting assay. The scale bar is 0.5 mm (N = 3). (*p < 0.05, **p < 0.01, ***p < 0.001).

As the possible oncogene in PDAC, we paid more attention of this target gene on PDAC malignant behavior. To underly the impact of RASAL2 on PDAC, we performed RASAL2 knockdown in PANC-1 cells with relatively high expression of RASAL2 in mRNA and protein level (**Fig 2a and Supplement Fig. 3a**). The inhibition of RASAL2 significantly decreased the cell proliferation as determined by MTT assay and colony formation assay (**Fig. 2b** and **2c**). Flow cytometry suggested that RASAL2 knockdown inhibited cell growth by inducing cell cycle arrest at G2/M phase in PANC-1 cells compared with control group (**Fig. 2d**). The ability of cell migration and invasion was attenuated by RASAL2 knockdown in cell migration and Matrigel transwell invasion assays (**Fig. 2e**). Similarly, in scratch wound healing assay, RASAL2 knockdown delayed wound healing, also indicating the reduction of cell motility (**Fig. 2f**). To observe the biology related gene alteration, western blot analyses showed that RASAL2 silence upregulated E-cadherin and Claudin1, and downregulated Vimentin and MMP2 (**Fig. 2g and Supplement Fig. 3b**). Therefore, RASAL2 plays an important role in maintaining malignant behaviors of PDAC cells.

**Figure 3:**
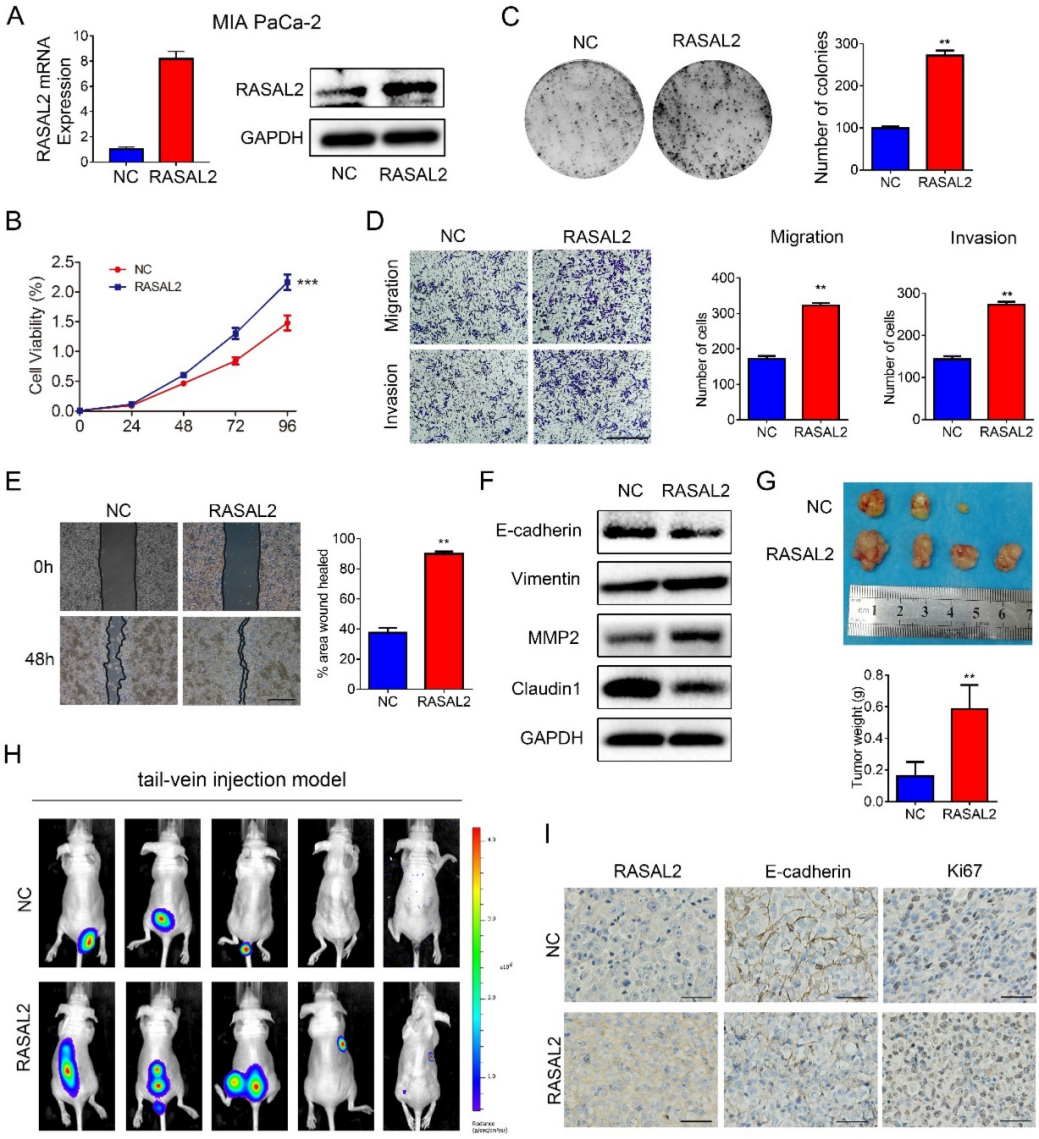
**Overexpression of RASAL2 promoted the malignancy of PDAC cells**. **a** Quantitative real-time RT-PCR and western blotting analysis of RASAL2 mRNA and protein expression in MIA PaCa-2 cells transfected with RASAL2 lentivirus and negative control. **b** and **c** MTT and colony formation assays, RASAL2 overexpression promoted the cell proliferation (right panel, quantitative analyses).** d** Representative images of Transwell migration and invasion assays in RASAL2 overexpressed MIA PaCa-2 subline (right, quantification analysis; N = 3). **e** Representative pictures of wound healing in MIA PaCa-2 cell line with overexpressing RASAL2 (right, quantification analysis; N = 3). **f** E-cadherin, Vimentin, MMP2 and Claudin1 were detected in cells overexpressing RASAL2 by western blot. **g** Image of xenograft tumors in nude mice inoculated subcutaneously with MIA PaCa-2 cells overexpressing RASAL2 or control cells (right panel, the mean tumor weights in histogram). **h** BLI images of athymic BALB/c nude mice injected via tail-vein with MIA PaCa-2/NC and MIA PaCa-2/RASAL2 cells. **i** Representative images of IHC staining of RASAL2, E-cadherin and Ki67 in subcutaneous tumors. 18S was used as the endogenous control for quantitative real-time RT-PCR, and GAPDH was used as a loading control for western blotting assay. The scale bar is 0.5 mm. (N = 3) (**p < 0.01).

Furthermore, RASAL2-overexpressing or negative control lentivirus were transfected stably into MIA PaCa-2 cells, and ectopic expression of RASAL2 was determined (**Fig. 3a and Supplement Fig.3c**). As compared with negative control cells, RASAL2 overexpression increased tumor cell viability (**Fig. 3b**) and the clonogenicity (**Fig. 3c**). Ectopic expression of RASAL2 enhanced the migration and invasion ability in MIA PaCa-2 cells as determined by transwell assay and scratch wound healing assay (**Fig. 3d** and **3e**). In MIA PaCa-2 cells with stably expressing RASAL2, expressions of E-cadherin and Claudin1 were decreased, whereas expressions of Vimentin and MMP2 were increased (**Fig. 3f and Supplement Fig.3d**).

In nude mouse models, RASAL2-overexpressing tumor cells and negative control cells were injected into flanks subcutaneously or tail vein intravenously. MIA PaCa-2 overexpressing RASAL2 formed larger size of tumors and developed more organ metastasis to lungs, livers, abdominal cavities and spinal columns than the control cells (**Fig. 3g** and** 4h**). Ki67 staining was increased in xenograft tumor tissues of MIA PaCa-2 overexpressing RASAL2 and E-cadherin staining was decreased (**Fig. 3i**).

### RASAL2 positively regulated the Hippo/YAP signaling

**Figure 4:**
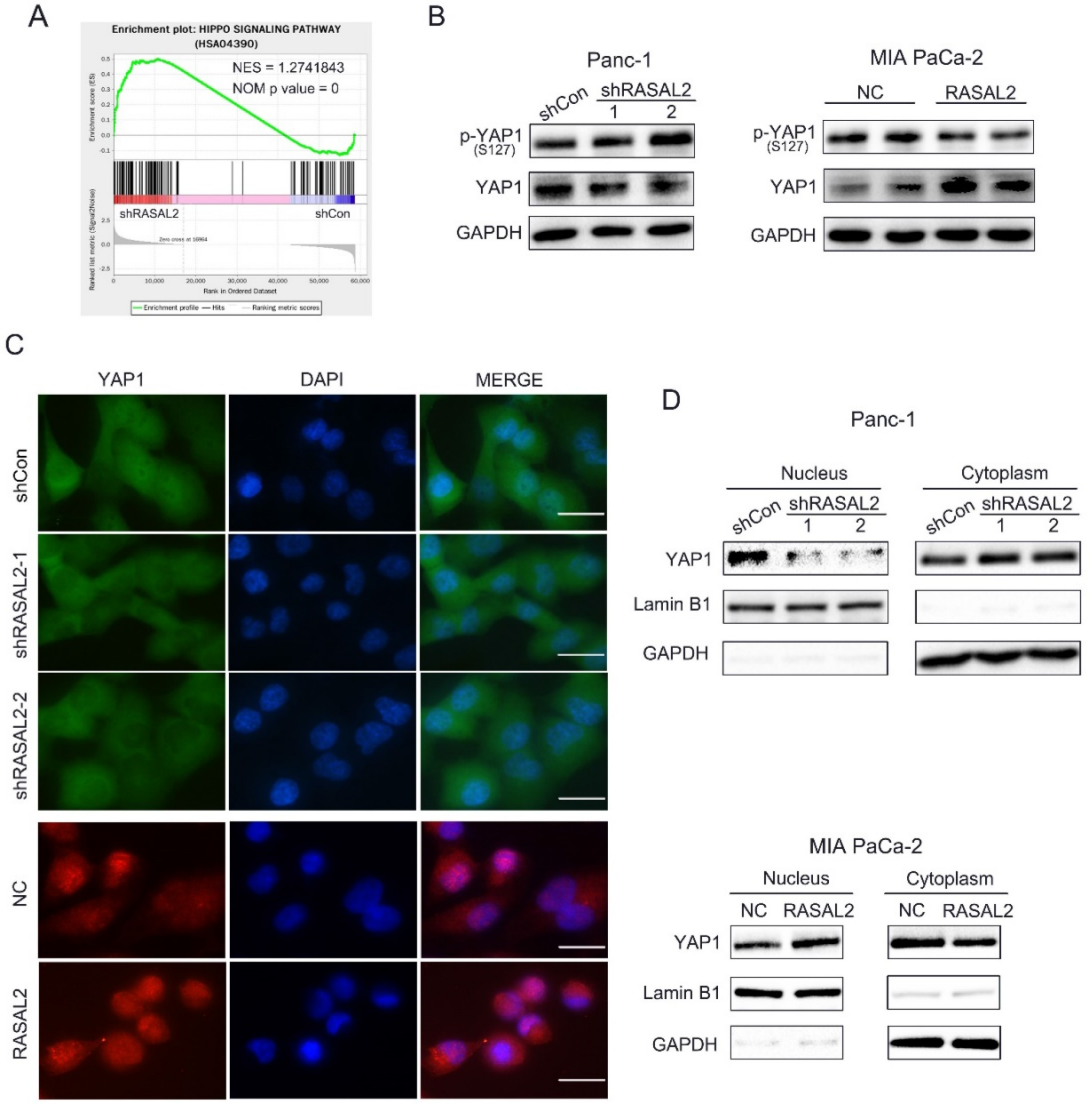
** RASAL2 regulated the Hippo/YAP signaling. a** Gene set enrichment analysis of Hippo pathway in PANC-1 cells with RASAL2 knockdown. **b** Western blot analysis showing the p-YAP1 (s127) and YAP1 expression after altered RASAL2 expression in PANC-1 and MIA PaCa-2 cells (N = 3). **c** The subcellular distribution of YAP1 was shown by immunofluorescence. The staining of YAP1 was green in PANC-1 cells with RASAL2 silencing and it was red in MIA PaCa-2 cells overexpressing RASAL2. Nuclei were shown as blue (N = 3). **d** The subcellular distributions of YAP1 in PANC-1 cells with RASAL2 knockdown and in MIA PaCa-2 cells overexpressing RASAL2 were determined by western blot analyses using the cytoplasmic and nuclear extracts (N = 3). 18S was used as the endogenous control for quantitative real-time RT-PCR, GAPDH and Lamin B1 were used as a loading control for western blotting assays. The scale bar is 20 µm.

Since the oncogenic role of RASAL2 was confirmed, the next step was to clarify the possible mechanism.To identify the target genes and signaling pathways of RASAL2, we conducted the RNA-seq analyses to determine the DEGs using total RNA from RASAL2-silenced PANC-1 cells and RASAL2-overexpressing MIA PaCa-2 cells.Based on the GESA of pathways in DEGs of PNAC-1 cells with RASAL2 silence, we observed a direct relationship between RASAL2 and Hippo pathway (**Fig. 4a**). Here we speculated that RASAL2 possibly regulated the Hippo signaling. To prove our hypothesis, we made a series of experiments. Western blot analysis revealed that knockdown of RASAL2 induced YAP1 phosphorylation (S127) and decreased YAP1 expression in PANC-1 cells. In contrast, upregulation of RASAL2 inhibited YAP1 phosphorylation (S127) and increased YAP1 expression (**Fig. 4b and Supplement Fig. 4a, b**). To observe the change of YAP1 staining by immunofluorescence we interesting found that hat distribution of YAP1 in nucleus was reduced in RASAL2-downregulated PANC-1 cells and was increased in RASAL2-upregulated MIA PaCa-2 cells (**Fig. 4c).** Additionally, as shown in **Fig. 4d and Supplement Fig. 3c, d**, western blot analysis of nucleus and cytoplasm extracts demonstrated that expression of YAP1 in nucleus was reduced in RASAL2-downregulated PANC-1 cells and was increased in RASAL2-upregulated MIA PaCa-2 cells. It was confirmed that the expression and location of YAP1 were dependent on RASAL2, which considered that RASAL2 could positively regulate Hippo/YAP1 signalling.

### RASAL2 increased TIAM1 expression through Hippo/YAP signaling

**Figure 5:**
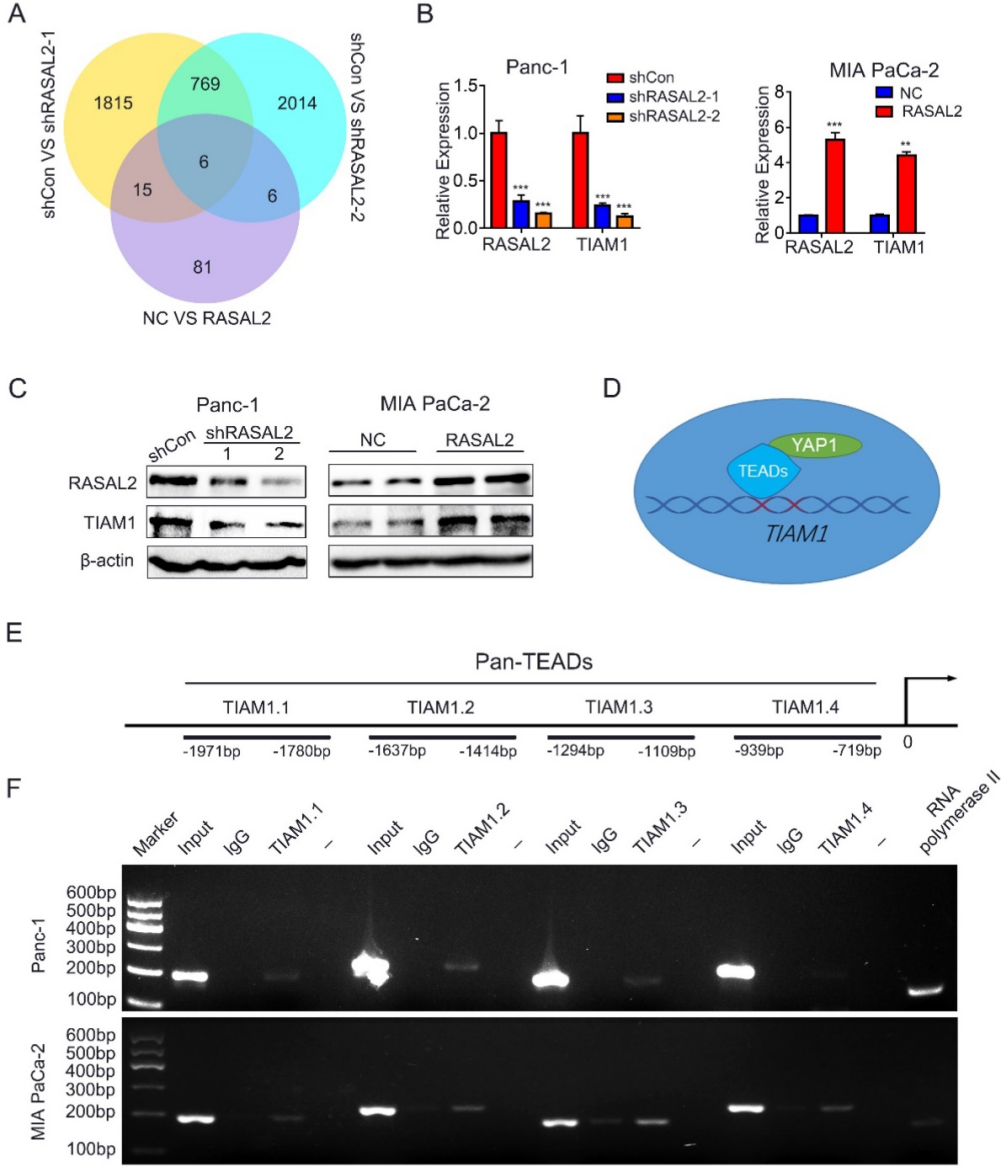
** RASAL2 increased TIAM1 expression through Hippo/YAP signaling**. **a** Venn diagram showing the differentially expressed genes in PANC-1 cells with RASAL2 silencing or MIA PaCa-2 cells overexpressing RASAL2.** b** Real-time quantitative PCR analyses of RASAL2 and TIAM1 mRNA expression in PANC-1 and MIA PaCa-2 cells with altered RASAL2 expression (N = 3). **c** Western blot analyses of TIAM1 protein expression in PANC-1 cells with RASAL2 silencing and in MIA PaCa-2 cells with RASAL2 overexpression (N = 3). **d and e** The schematic diagram of TIAM1 promoter. **f** Chromatin immunoprecipitation (ChIP) assay: The binding sites of Pan-TEADs on the TIAM1 promoter were detected by PCR in PANC-1 and MIA PaCa-2 cells. RNA polymerase II was used as the positive control. 18S was used as the endogenous control for quantitative real-time RT-PCR, β-actin was used as a loading control for western blotting assays. The scale bar is 20 µm.

To find the target gene of Hippo/YAP1 signalling, we screened the candidate genes from RNA-seq results, and six vital genes were identified (**Fig. 5a**). To verify these target genes, we examined the RNA-seq results with qRT-PCR and obtained the significant difference (**Supplement Fig. 5a**). Since the expression of TIAM1, one of the six crucial genes, directly correlated with malignant progression of various tumors, and appears to be the protein that interacts directly with RASAL2 as predicted by BioGRID 4.2 (https://thebiogrid.org/). We consider TIAM1 as our target gene. Indeed, the mRNA and protein expressions of TIAM1 were positively correlated with those of RASAL2 (**Fig. 5b, c and Supplement Fig. 5b**).

To determine whether YAP1, as a transcriptional coactivator of TEADs transcription factors, mediated the regulation of TIAM1 mRNA expression by RASAL2, the possible binding sites of TEADs (TEAD1-4) in the promoter of TIAM1 were predicted by JASPAR^2020^ (http://jaspar.genereg.net/). ChIP assay confirmed that Pan-TEAD could bind to TIAM1.1-TIAM1.3 sequence in PANC-1 cells and TIAM1.1-TIAM1.4 sequence in MIA PaCa-2 cells (**Fig. 5d, 5e** and **5f**), indicating that RASAL2 promoted the TIAM1 transcription and increased its mRNA expression by upregulation of YAP1.

### RASAL2 accumulated TIAM1 protein expression with a direct bindig way and modulated the ubiquitin-proteasome pathway

**Figure 6:**
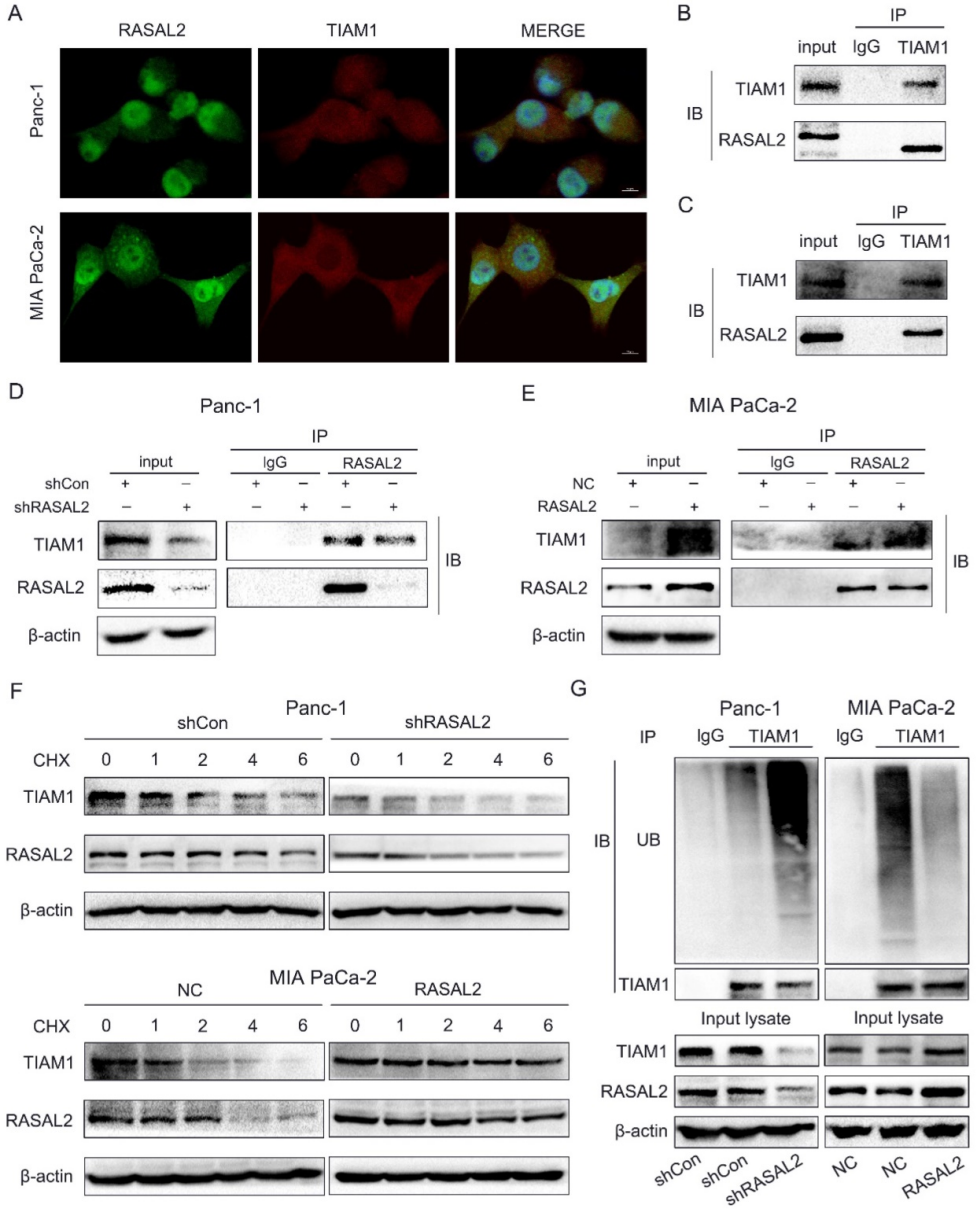
** RASAL2 may directly bind with TIAM1**. **a** Staining of RASAL2 and TIAM1 was performed by double immunofluorescence assay in PANC-1 and MIA PaCa-2 cells. **b** and **c** Co-immunoprecipitation (Co-IP) assay was used to detect the interaction between RASAL2 and TIAM1 in PANC-1 and MIA PaCa-2 cells. **d** and **e** The direct binding between RASAL2 and TIAM1 was shown by Co-IP assay in PANC-1 cells with RASAL2 silencing and MIA PaCa-2 cells with RASAL2 overexpression. **f** In PANC-1 cells with RASAL2 silencing and MIA PaCa-2 cells with RASAL2 overexpression were incubated with cycloheximide (100 μg/mL) for 1, 2, 4 and 6 h. Cell lysates were prepared and used for TIAM1 and RASAL2 expression analyses by western blot. **g** PANC-1 cells with RASAL2 downregulation and MIA PaCa-2 cells with RASAL2 upregulation were pretreated with MG132 (20 μM) for 6h, the ubiquitination assay was performed by using immunoprecipitation and western blot analyses.

Interestingly, when we verified the relation between RASAL2 and YAP1/TIAM1, we observed that RASAL2 and TIAM1 could co-localize in the cells through double immunofluorescence assay (**Fig. 6a**). It seemed that RASAL2 could not only influence TIAM1 expression through Hippo/YAP1 signaling, but also directly bind with TIAM1 protein. This could increase the TIAM1 protein accumulation, which benefits for PDAC malignant behavior. To verify the hypothesis, we further performed the Co-IP assay. The direct interaction between RASAL2 and TIAM1 in PANC-1 cells (**Fig. 6b**) and MIA PaCa-2 cells (**Fig. 6c**) were observed. Additionally, inhibition of RASAL2 reduced the direct binding, while RASAL2 overexpression enhanced the interaction between RASAL2 and TIAM1 protein (**Fig. 6d** and** 6e**).

Since RASAL2 upregulated the expression of TIAM1 protein through a direct binding way, we further explored whether RASAL2 influenced the stability of TIAM1. PANC-1 and MIA PaCa-2 cells were treated with protease inhibitor MG132 (20 μM) and lysine autophagy inhibitor chloroquine (20 μM), then cycloheximide (CHX, 100 μg/mL) was added for 1, 2, 4 and 6 h. Western blot was used to examine the TIAM1 protein expression. As shown in **Supplement Fig. 6a**, TIAM1 degradation was blocked by MG132, but not by chloroquine, suggesting that TIAM1 degraded mainly via ubiquitin-proteasome pathway. Furthermore, PANC-1 cells with RASAL2 knockdown and MIA PaCa-2 cells were treated with CHX (100 μg/mL) for 1, 2, 4 and 6 h,and TIAM1 protein levels were detected by western blot analyses. As shown in **Fig. 6f and Supplement Fig. 6b**, silencing RASAL2 accelerated TIAM1 degradation, while overexpressing RASAL2 attenuated TIAM1 degradation. To confirm this result, we performed the ubiquitination assay with modified immunoprecipitation. Our results showed that ubiquitination of TIAM1 was increased by silencing RASAL2, but decreased by RASAL2 overexpression. Therefore, the ubiquitin-proteasome pathway was mainly responsible for TIAM1 protein degradation in PDAC cells.Therefore, RASAL2 not only transactivated TIAM1 expression, but also bound directly with TIAM1 protein. Finally, RASAL2 expression was positively correlated with TIAM1 expression at both mRNA and protein levels.

### RASAL2 promoted PDAC malignancy by enhancing YAP1/TIAM1 signaling

**Figure 7:**
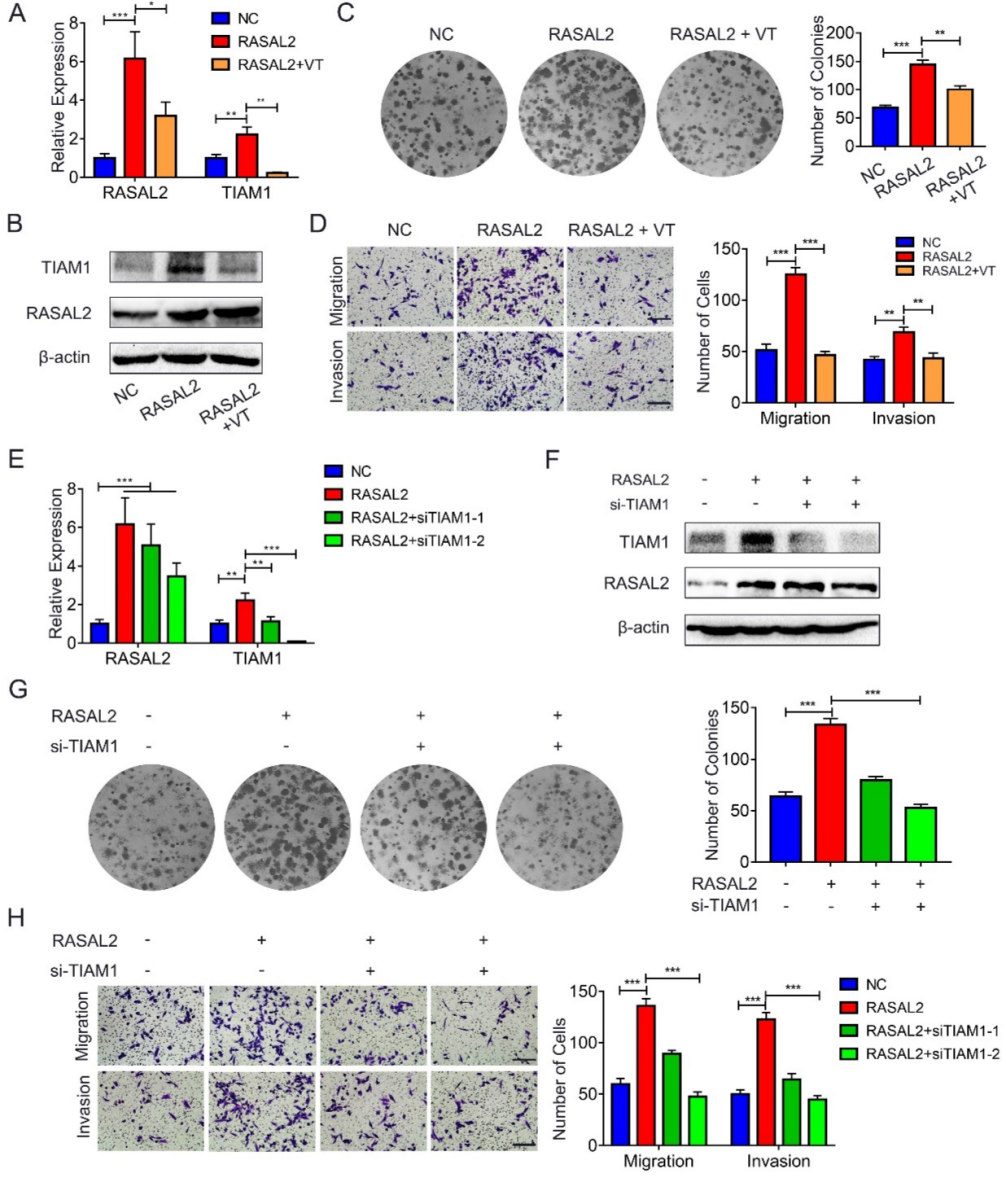
**RASAL2 enhanced YAP1/TIAM1 signaling and promoted the malignancy of PDAC cells**. **a** and **b** MIA PaCa-2 cells overexpressing RASAL2 weretreated with verteporfin (3 μM) for 24h or 48h, Quantitative real-time RT-PCR and western blotting analysis were used to determine the mRNA and protein expression of TIAM1. **c** and **d** Representative images of colony formation assay and transwell migration and invasion assays: MIA PaCa-2 cells with RASAL2 overexpression were incubated with verteporfin. **e** and **f** TIAM1 was knocked down by siRNA in RASAL2-overexpressing MIA PaCa-2 cells, and the expression of mRNA and protein TIAM1 were examined by Quantitative real-time RT-PCR and western blotting analyses. **g** and **h** Representative images of colony formation assay, Transwell migration and invasion assays using RASAL2-overexpressing MIA PaCa-2, in which TIAM1 was silenced by siRNA.

To determine further whether RASAL2 promoted mRNA expression of TIAM1 by upregulating YAP1, RASAL2-overexpressing MIA PaCa-2 cells were treated with verteporfin, a pharmacological YAP1 inhibitor. qRT-PCR and western blot analyses showed that both mRNA and protein expression of TIAM1 decreased in the cells treated with verteporfin (**Fig. 7a** and **7b and Supplement Fig. 7a**). Similarly, the colony formation assay and transwell migration or invasion assay revealed that the tumor cells proliferation, migration and invasion were inhibited by verteporfin treatment (**Fig. 7c** and **7d**). Conversely, knockdown of TIAM1 in RASAL2-overexpressing MIA PaCa-2 cells with siRNA reduced the ability of tumor cell proliferation, migration and invasion (**Fig. 7e** - **7h and Supplement Fig. 7b**). Consistently, TIAM1 mRNA expression was increased in PDAC of TCGA matched GTEx database (**Supplement Fig. 8a**), Finally, RASAL2 expression was positively correlated with TIAM1 expression at both mRNA and protein levels (**Supplement Fig. 8b** and **c**). Therefore, RASAL2 increased TIAM1 expression and promoted the malignancy of tumor cells by upregulation of YAP1.

## Discussion

Patients with pancreatic cancer often develop distant metastasis at the time of diagnosis, and have no chance to undergo surgery 2-4. The continuous increase burden of pancreatic cancer all over the world will sustain due to ever-increasing of aging population and exposure to many risk factors 19-21. Better understanding the mechanism of PDAC tumorigenesis and metastasis is essential for early diagnosis and effective treatment of this deadly disease. In this study, we firstly demonstrated that RASAL2 expression was significantly increased at both mRNA and protein levels in PDAC cells and tissue specimens as compared to that in normal pancreatic tissues. High expression of RASAL2 predicted a poor prognosis in PDAC patients. RASAL2 promoted tumor cell proliferation, migration and invasion in PDAC cells* in vitro* and growth and metastasis in animal models. Mechanistically, RASAL2 inhibited YAP1 phosphorylation, increased TIAM1 mRNA expression and suppressed ubiquitination of TIAM1 protein. Therefore, RASAL2 enhances YAP1/TIAM1 signaling and promotes PDAC development and progression, thus serving as a potential therapeutic target.

RASAL2 has diverse functions and its contradictory roles in tumor development and progression is controversial and appears to be dependent on the cell context or tumor types 7, 13. Early studies identified RASAL2 as a tumor suppressor, with a notion mostly inferring from findings on tumor angiogenesis and metastasis. For examples, in luminal B breast cancer, lung adenocarcinoma, ovarian cancer, nasopharyngeal carcinoma and bladder cancer, RASAL2 inhibits tumor cell migration and invasion by suppressing RAS signaling pathway and epithelial mesenchymal transition (EMT) 8, 9, 22-24. In renal cell carcinoma and bladder cancer, RASAL2 suppresses angiogenesis by activating GSK3β or inhibiting AKT pathway 10, 11. RASAL2 also suppresses cancer progression via the RAS-ERK pathway 22, phosphoinositide 3-kinase (PI3K)/AKT/mechanistic target of rapamycin (mTOR) signaling pathway 7, nuclear factor (NF)-κB pathway 7, and ERK/mitogen activated protein kinase (MAPK) pathway 8. However, RASAL2 also functions as an oncogene in various cancers via the Hippo signaling pathway 16, WNT/β-catenin pathway 25, and RAS signaling pathway 18. Recent studies showed that RASAL2 promoted malignancy of tumor cells. A genome-wide study indicated that RASAL2 downregulation inhibited tumor cell growth and metastasis in liver cancer and triple-negative breast cancer 14, 25. The YAP1 signaling pathway was activated by RASAL2 to promote proliferation and metastasis of tumor cells in colorectal cancer, regardless of KRAS mutation status 16. However, little is know about this gene on pancreatic cancer progression. In our current study, we found Aberrant expression of RASAL2 was increased in PDAC and predicted poor prognosis. Knockdown of RASAL2 suppressed malignant behaviors of PDAC cells in vitro. It is confirmed that RASAL2 could play an oncogenic role on PDAC malignant behavior.

Currently, the specific mechanisms for pro- or anti-oncogenic behaviors of RASAL2 are unknown. Apparently, phosphorylated RASAL2 facilitates breast cancer progression and the ratio of p-RASAL2/non-p-RASAL2 is one of the determining factors for the effect of RASAL2 in ER^+^ and ER^-^ breast cancer 17, 26. Our current study identified a novel mechanism, where RASAL2 influenced Hippo pathway by accelerating YAP1 transportation to the nucleus. Hippo pathway components are structurally and functionally conserved and have diverse functions, including development and tumorigenesis. The core components of the Hippo pathway are highly conserved and consist of a kinase cascade and downstream transcription factors 27. In mammals, the kinase cascade includes mitogen-activated protein kinase (MAP4K) family, mammalian STE20 like kinase 1/2 (MST1/2), and large tumor suppressor kinase 1/2 (LATS1/2), while the downstream effectors are further grouped into DNA-binding protein TEAD 1/2/3/4 andYAP1/TAZ transcription coactivators 27. TIAM1 interacts with TAZ in the cytoplasm to promote TAZ degradation by the destruction complex, whereas it antagonizes binding of TAZ/YAP to TEAD in the nucleus in colorectal cancer 28, 29.

To clarify the target gene for RASAL2, we applied RNA-seq for the detection. TIAM1 alteration was considered as a target gene. TIAM1 was initially found in mouse T lymphoma cells and then confirmed as a key gene associated with cancer malignancy 30-35. In PDAC, high expression of TIAM1 suggested a poor prognosis and silencing TIAM1 decreased tumor cell proliferation, migration and invasion 36. Moreover, a systematic review and meta-analysis revealed that high TIAM1 expression was significantly related with shorter overall survival (OS) and disease-free survival (DFS) and was associated with metastasis in patients with solid cancers 37, 38. PDAC expresses a high level of TIAM1 expression and predicts a poor prognosis, while mechanistically silencing TIAM1 decreases tumor cell proliferation, migration and invasion 36. Therefore, in PDAC, high level of RASAL2 might cause TIAM1 overexpression. Consistently, TIAM1 acts as a key gene associated with cancer malignancy 30-34, high TIAM1 expression was significantly related with shorter overall survival (OS) and disease-free survival (DFS) and was clearly associated with metastasis in patients with solid cancers 37, 38.Our current study has for the first time shown that RASAL2 promotes PDAC malignancy by enhancing YAP1/TIAM1 signaling in PDAC. Specifically, RASAL2 promoted TIAM1 expression by upregulating YAP1. Thus, TIAM1 is intimately involved in Hippo signaling and play an important role in carcinogenesis. The mechanisms responsible for the upregulation of YAP1 by RASAL2 warrant further investigations.

## Conclusion

**Figure 8:**
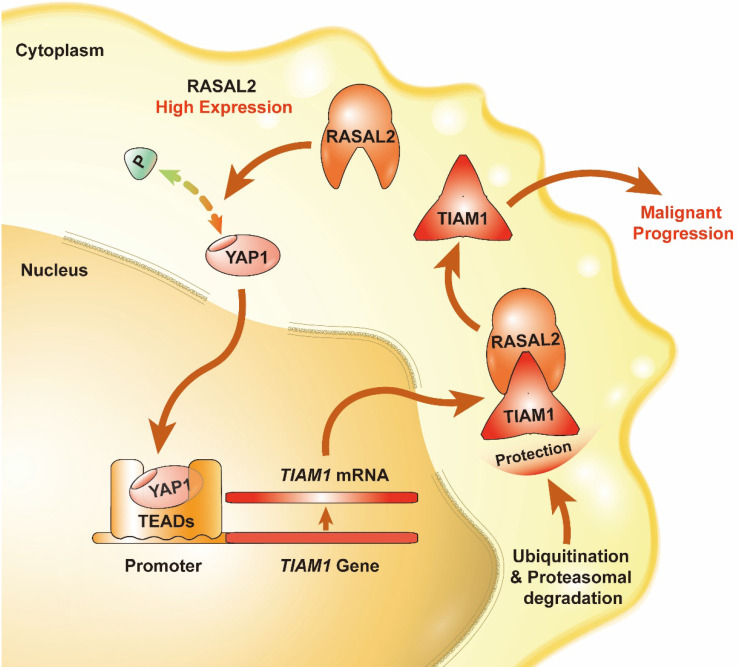
Schematic of the RASAL2-regulated YAP1/TIAM1 pathway in PDAC.

In summary, we found that the expression of RASAL2 increased in PDAC as compared to normal pancreatic tissues, promoted tumor cell proliferation, migration and invasion, and high expression of RASAL2 predicted a poor prognosis in PDAC patients. Mechanistically, as shown in** Figure 8**, RASAL2 inhibited YAP1 phosphorylation, increased TIAM1 mRNA expression and suppressed ubiquitination of TIAM1 protein, thus enhancing YAP1/TIAM1 signaling and promoting PDAC development and progression. Therefore, RASAL2 could be a potential novel biomarker of diagnosis and prognosis and target for pancreatic cancer treatment.

## Supplementary Material

Supplementary figures and table.

## Abbreviations

PDAC: Pancreatic ductal adenocarcinoma
RAS-GAP: RAS GTPase-activating protein
PC: pancreatic cancer
GEO: Gene Expression Omnibus
DEGs: differentially expressed genes
TCGA: The Cancer Genome Atlas
OS: overall survival
DFS: disease-free survival
RNA-seq: RNA sequencing
GSEA: Gene Set Enrichment Analysis
EMT: epithelial mesenchymal transition
CBTCCCAS: Chinese Academy of Sciences Cell Bank of Type Culture Collection
DMEM: Dulbecco's modified Eagle's medium
FBS: foetal bovine serum
PBS: phosphate-buffered saline

## References

[1] Grossberg AJ, Chu LC, Deig CR, Fishman EK, Hwang WL, Maitra A (2020). Multidisciplinary standards of care and recent progress in pancreatic ductal adenocarcinoma. CA Cancer J Clin.

[2] Siersema PD (2019). Pancreatic Cancer Awareness Issue 2019. Endoscopy.

[3] Neoptolemos JP, Stocken DD, Friess H, Bassi C, Dunn JA, Hickey H (2004). A randomized trial of chemoradiotherapy and chemotherapy after resection of pancreatic cancer. N Engl J Med.

[4] Conroy T, Hammel P, Hebbar M, Ben Abdelghani M, Wei AC, Raoul JL (2018). FOLFIRINOX or Gemcitabine as Adjuvant Therapy for Pancreatic Cancer. N Engl J Med.

[5] Maertens O, Cichowski K (2014). An expanding role for RAS GTPase activating proteins (RAS GAPs) in cancer. Adv Biol Regul.

[6] Weeks A, Okolowsky N, Golbourn B, Ivanchuk S, Smith C, Rutka JT (2012). ECT2 and RASAL2 mediate mesenchymal-amoeboid transition in human astrocytoma cells. Am J Pathol.

[7] McLaughlin SK, Olsen SN, Dake B, De Raedt T, Lim E, Bronson RT (2013). The RasGAP gene, RASAL2, is a tumor and metastasis suppressor. Cancer cell.

[8] Hui K, Gao Y, Huang J, Xu S, Wang B, Zeng J (2017). RASAL2, a RAS GTPase-activating protein, inhibits stemness and epithelial-mesenchymal transition via MAPK/SOX2 pathway in bladder cancer. Cell Death Dis.

[9] Olsen SN, Wronski A, Castano Z, Dake B, Malone C, De Raedt T (2017). Loss of RasGAP Tumor Suppressors Underlies the Aggressive Nature of Luminal B Breast Cancers. Cancer Discov.

[10] Hui K, Wu S, Yue Y, Gu Y, Guan B, Wang X (2018). RASAL2 inhibits tumor angiogenesis via p-AKT/ETS1 signaling in bladder cancer. Cellular signalling.

[11] Hui K, Yue Y, Wu S, Gu Y, Guan B, Wang X (2018). The expression and function of RASAL2 in renal cell carcinoma angiogenesis. Cell Death Dis.

[12] Wang Z, Wang J, Su Y, Zeng Z (2015). RASAL2 inhibited the proliferation and metastasis capability of nasopharyngeal carcinoma. Int J Clin Exp Med.

[13] Shen J, Wang Y, Hung MC (2013). RASAL2: wrestling in the combat of Ras activation. Cancer cell.

[14] Feng M, Bao Y, Li Z, Li J, Gong M, Lam S (2014). RASAL2 activates RAC1 to promote triple-negative breast cancer progression. The Journal of clinical investigation.

[15] Fang JF, Zhao HP, Wang ZF, Zheng SS (2017). Upregulation of RASAL2 promotes proliferation and metastasis, and is targeted by miR-203 in hepatocellular carcinoma. Mol Med Rep.

[16] Pan Y, Tong JHM, Lung RWM, Kang W, Kwan JSH, Chak WP (2018). RASAL2 promotes tumor progression through LATS2/YAP1 axis of hippo signaling pathway in colorectal cancer. Mol Cancer.

[17] Wang X, Qian C, Yang Y, Liu MY, Ke Y, Qian ZM (2019). Phosphorylated Rasal2 facilitates breast cancer progression. EBioMedicine.

[18] Zhang W, Lu Y, Li X, Zhang J, Lin W, Zhang W (2019). IPO5 promotes the proliferation and tumourigenicity of colorectal cancer cells by mediating RASAL2 nuclear transportation. J Exp Clin Cancer Res.

[19] Rahib L, Smith BD, Aizenberg R, Rosenzweig AB, Fleshman JM, Matrisian LM (2014). Projecting cancer incidence and deaths to 2030: the unexpected burden of thyroid, liver, and pancreas cancers in the United States. Cancer research.

[20] Bray F, Ferlay J, Soerjomataram I, Siegel RL, Torre LA, Jemal A (2018). Global cancer statistics 2018: GLOBOCAN estimates of incidence and mortality worldwide for 36 cancers in 185 countries. CA Cancer J Clin.

[21] Siegel RL, Miller KD, Jemal A (2020). Cancer statistics, 2020. CA Cancer J Clin.

[22] Huang Y, Zhao M, Xu H, Wang K, Fu Z, Jiang Y (2014). RASAL2 down-regulation in ovarian cancer promotes epithelial-mesenchymal transition and metastasis. Oncotarget.

[23] Li N, Li S (2014). RASAL2 promotes lung cancer metastasis through epithelial-mesenchymal transition. Biochemical and biophysical research communications.

[24] Sears R, Gray JW (2017). Epigenomic Inactivation of RasGAPs Activates RAS Signaling in a Subset of Luminal B Breast Cancers. Cancer Discov.

[25] Stefanska B, Cheishvili D, Suderman M, Arakelian A, Huang J, Hallett M (2014). Genome-wide study of hypomethylated and induced genes in patients with liver cancer unravels novel anticancer targets. Clin Cancer Res.

[26] Galindo-Hernandez O (2020). Rasal2, highlighting the importance of phosphorylation on function in tumour development. EBioMedicine.

[27] Wu Z, Guan KL (2021). Hippo Signaling in Embryogenesis and Development. Trends Biochem Sci.

[28] Azzolin L, Piccolo S (2017). A TIAM Double Hit to Oppose YAP/TAZ. Cancer cell.

[29] Diamantopoulou Z, White G, Fadlullah MZH, Dreger M, Pickering K, Maltas J (2017). TIAM1 Antagonizes TAZ/YAP Both in the Destruction Complex in the Cytoplasm and in the Nucleus to Inhibit Invasion of Intestinal Epithelial Cells. Cancer cell.

[30] Mertens AE, Roovers RC, Collard JG (2003). Regulation of Tiam1-Rac signalling. FEBS Letters.

[31] Buongiorno P, Pethe VV, Charames GS, Esufali S, Bapat B (2008). Rac1 GTPase and the Rac1 exchange factor Tiam1 associate with Wnt-responsive promoters to enhance beta-catenin/TCF-dependent transcription in colorectal cancer cells. Mol Cancer.

[32] Viaud J, Lagarrigue F, Ramel D, Allart S, Chicanne G, Ceccato L (2014). Phosphatidylinositol 5-phosphate regulates invasion through binding and activation of Tiam1. Nat Commun.

[33] Liu L, Wu B, Cai H, Li D, Ma Y, Zhu X (2018). Tiam1 promotes thyroid carcinoma metastasis by modulating EMT via Wnt/beta-catenin signaling. Exp Cell Res.

[34] Izumi D, Toden S, Ureta E, Ishimoto T, Baba H, Goel A (2019). TIAM1 promotes chemoresistance and tumor invasiveness in colorectal cancer. Cell Death Dis.

[35] Boissier P, Huynh-Do U (2014). The guanine nucleotide exchange factor Tiam1: a Janus-faced molecule in cellular signaling. Cellular signalling.

[36] Ding M, Li Y, Yang Y, Zhu K, Che S, Lin Z (2018). Elevated expression of Tiam1 is associated with poor prognosis and promotes tumor progression in pancreatic cancer. Onco Targets Ther.

[37] Yang C, Ma C, Li Y, Mo P, Yang Y (2019). High Tiam1 expression predicts positive lymphatic metastasis and worse survival in patients with malignant solid tumors: a systematic review and meta-analysis. Onco Targets Ther.

[38] Ding J, Yang F, Wu W (2019). Tiam1 high expression is associated with poor prognosis in solid cancers: A meta-analysis. Medicine (Baltimore).

