# Supplementary material for: RASAL2 mediated the enhancement of YAP1/TIAM1 signaling promotes malignant phenotypes of pancreatic ductal adenocarcinoma
Source: Int J Biol Sci. 2022 Jun 27;18(10):4245–59. doi: 10.7150/ijbs.72204 (PMC9274491; doi:10.7150/ijbs.72204)
Supplement: Supplementary file 1 — Supplementary figures and table. [file ijbsv18p4245s1.pdf]

Supplement Fig. 1

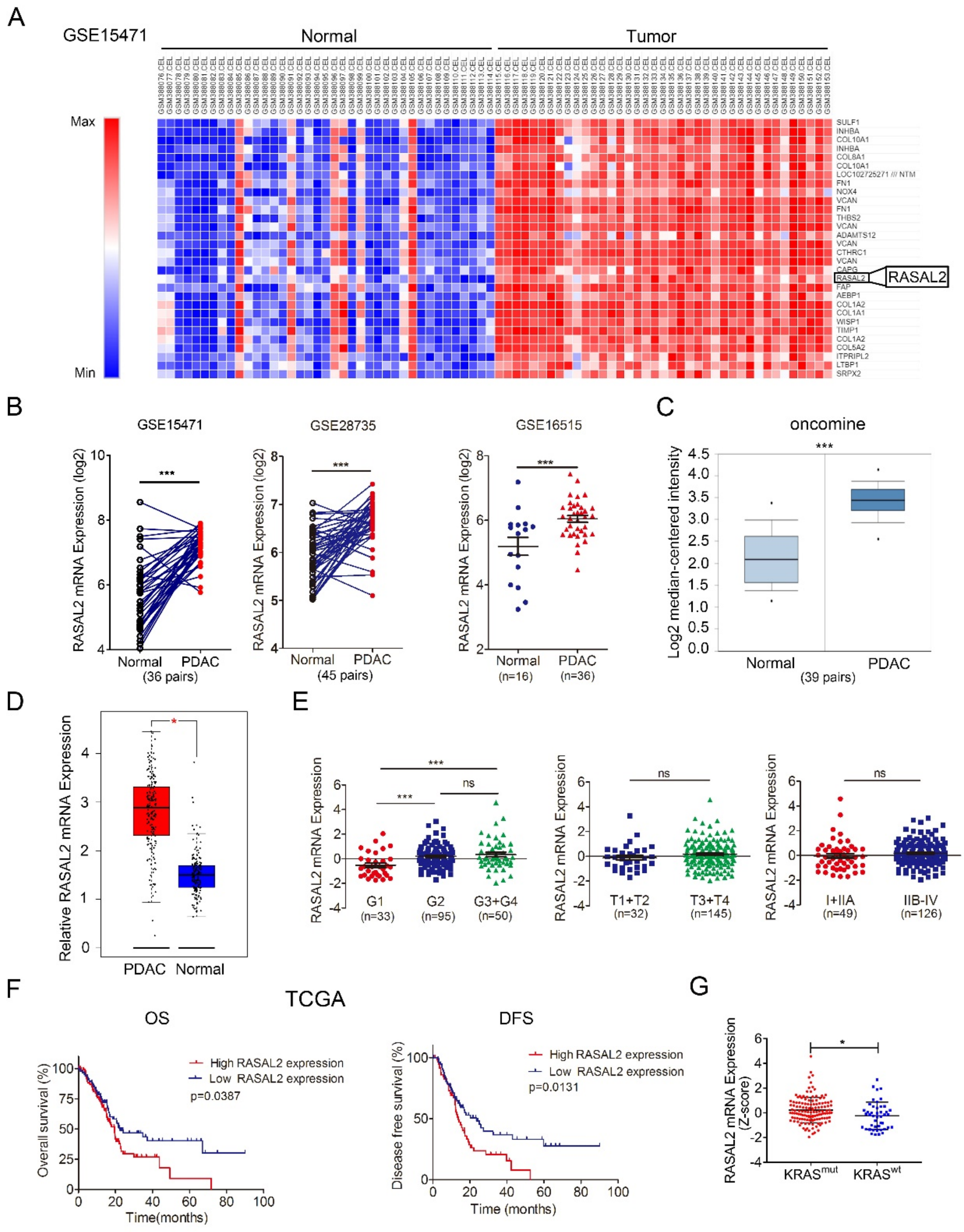

Supplement Fig. 2

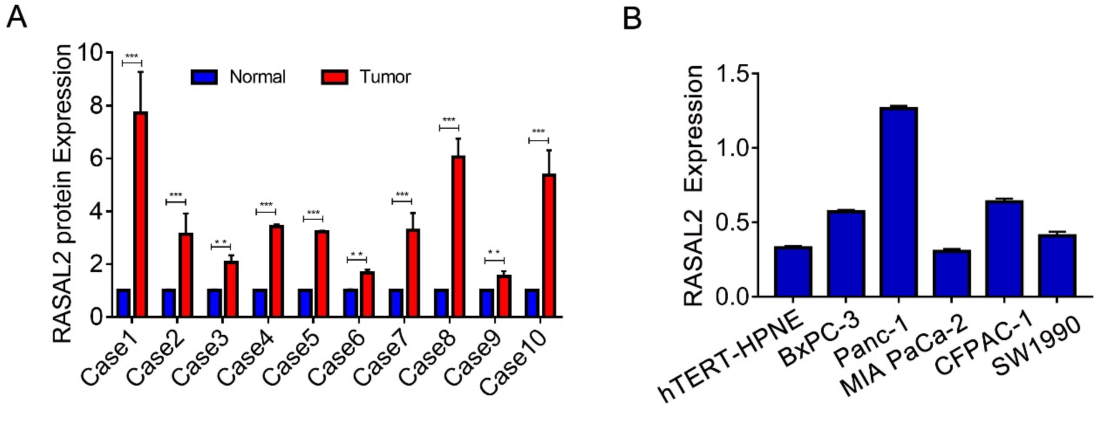

Supplement Fig. 3

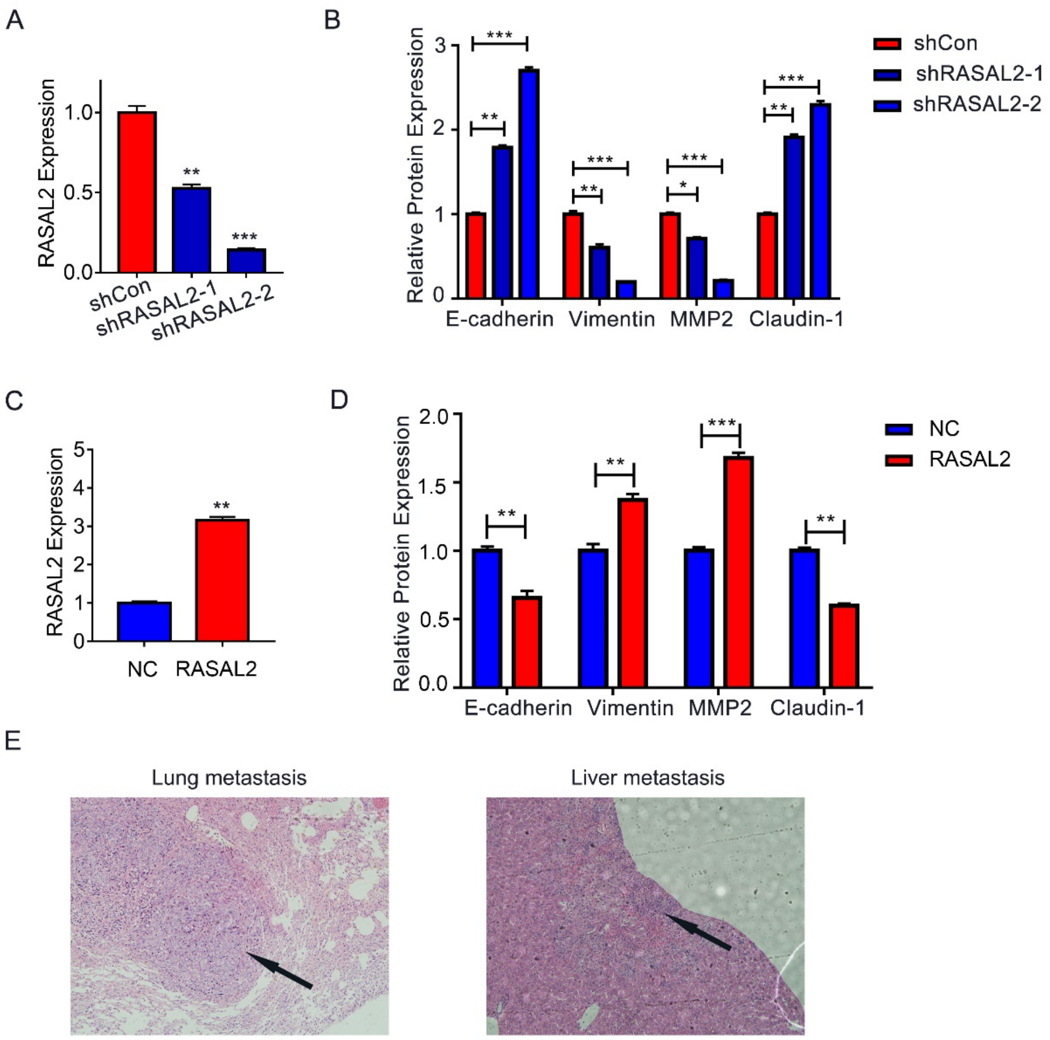

Supplement Fig. 4

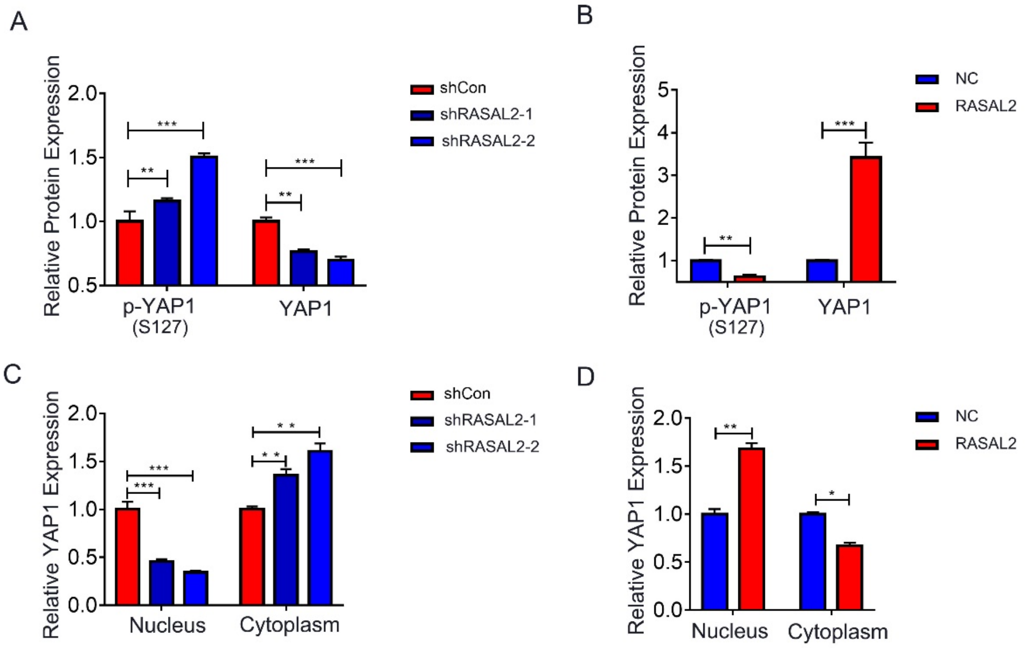

Supplement Fig. 5

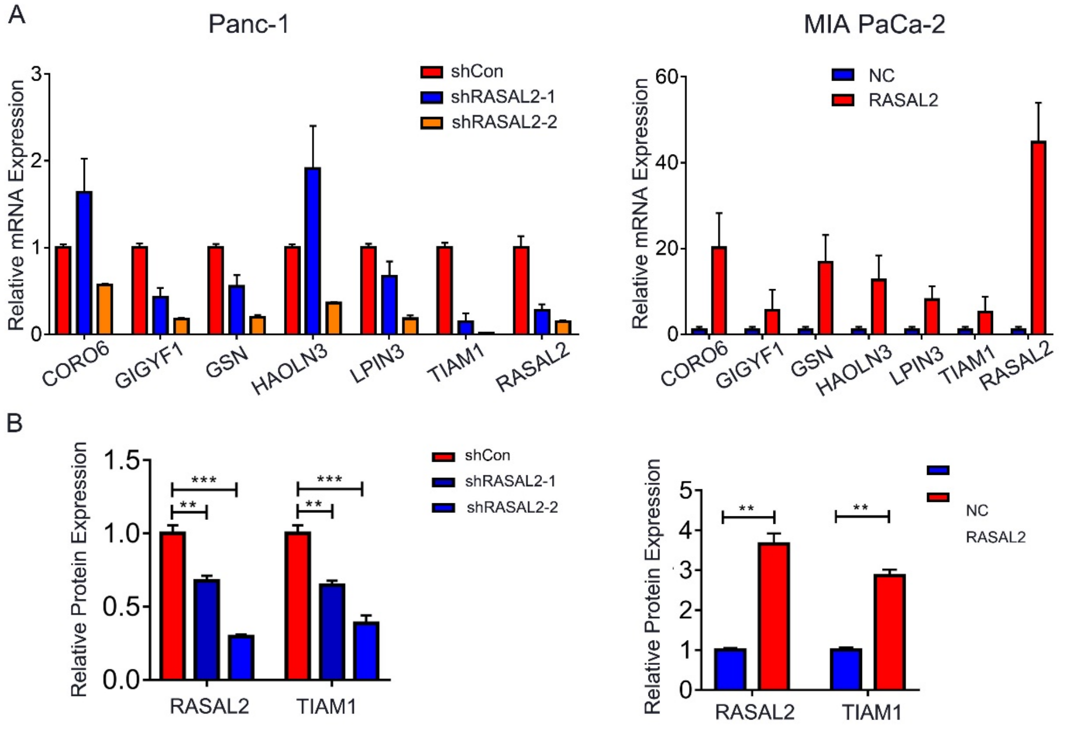

Supplement Fig. 6

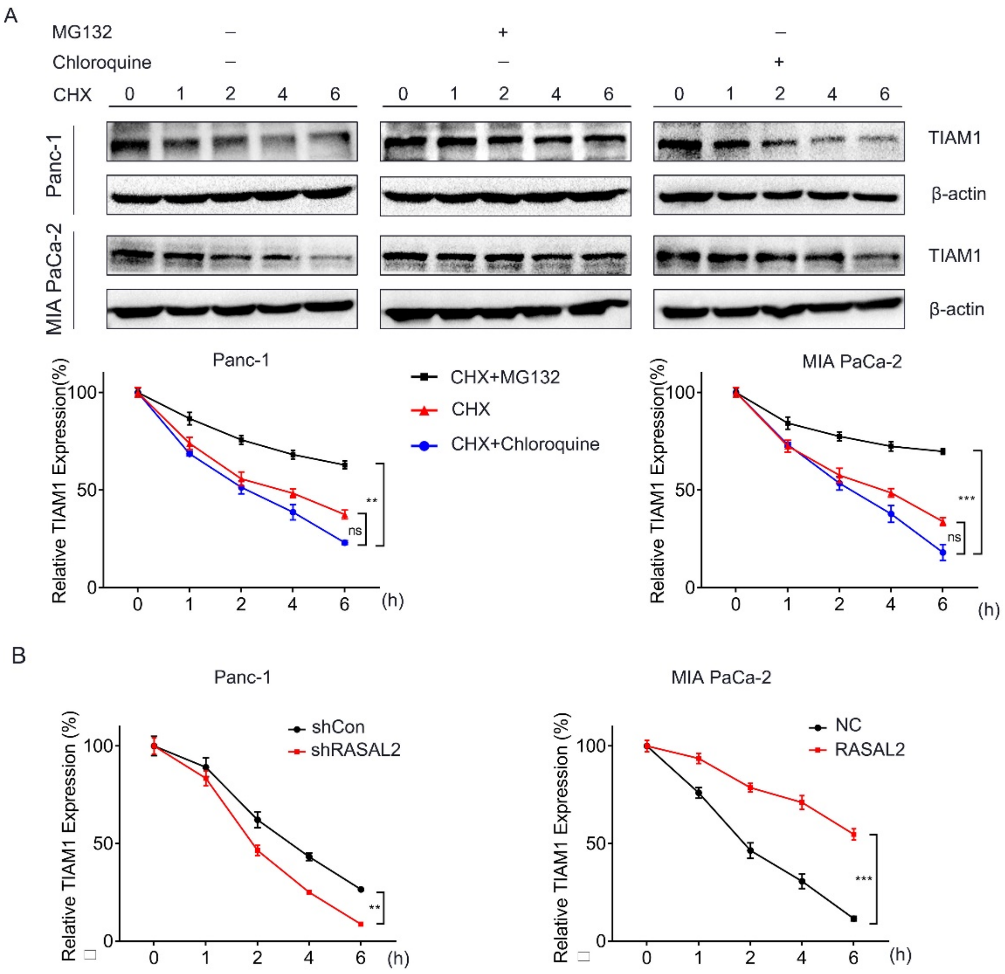

Supplement Fig. 7

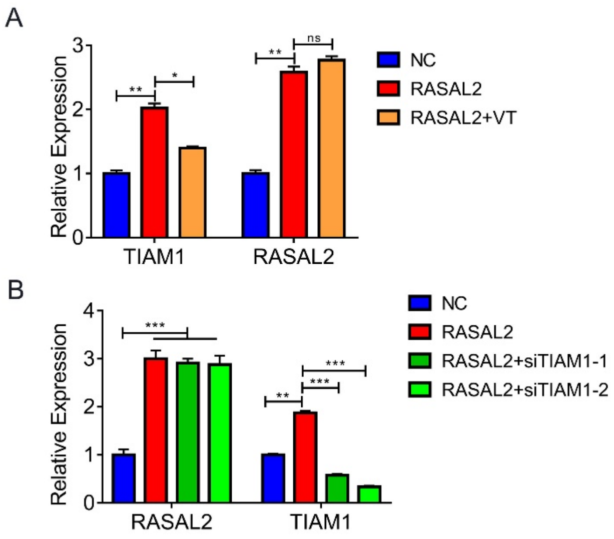

Supplement Fig. 8

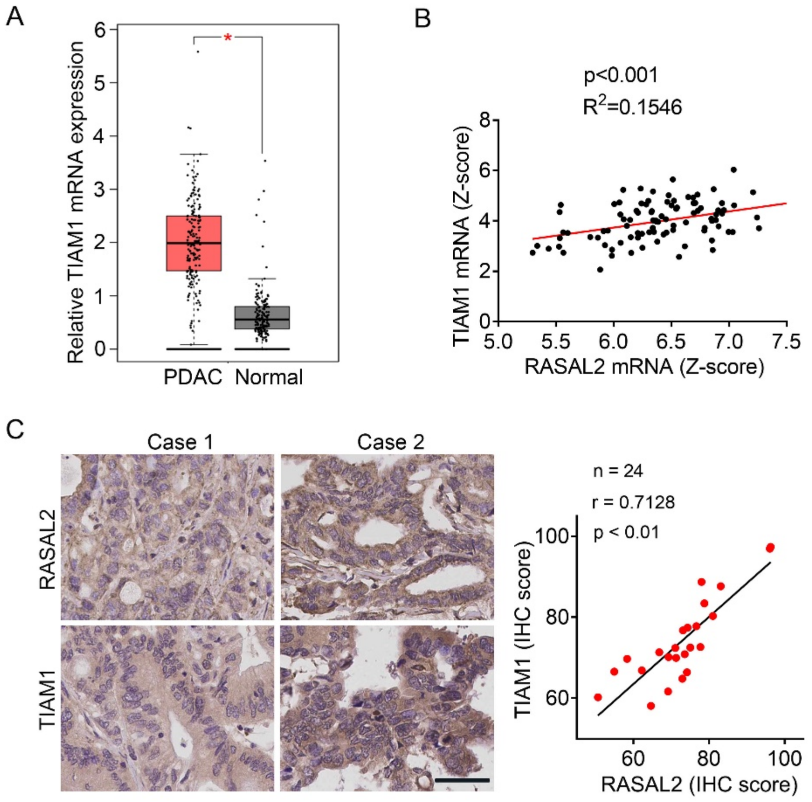

Supplement Table1

Expression of RASAL2 according to clinical features of 176 pancreatic cancer patients

Characteristic	High	Low	*P*-value
Age			
≥65 years	40	55	0.546
< 65 years	38	43	
Sex			
Male	42	54	0.88
Female	36	44	
Grade			
G1	6	24	0.01*
G2	48	46	
G3/4	24	25	
Stage			
I/IIA	20	29	0.502
IIB-IV	58	66	
Size			
≥ 4cm	39	30	0.017*
< 4cm	35	59	
New tumor event			
Yes	29	22	0.04*
NO	41	65	

*Statistical significant difference
